# Reduced Hippocampal Dentate Cell Proliferation and Impaired Spatial Memory Performance in Aged-Epileptic Rats

**DOI:** 10.3389/fneur.2013.00106

**Published:** 2013-07-26

**Authors:** Clarissa F. Cavarsan, Claudio M. Queiroz, Jair Guilherme dos Santos, Gilberto F. Xavier, Luiz Eugênio Mello, Luciene Covolan

**Affiliations:** ^1^Department of Physiology, Universidade Federal de São Paulo – UNIFESP, São Paulo, Brazil; ^2^Brain Institute, Universidade Federal do Rio Grande do Norte, Natal, Brazil; ^3^Faculdade de Ciências Médicas da Santa Casa de São Paulo – FCMSCSP, São Paulo, Brazil; ^4^Department of Physiology, Biosciences Institute, Universidade de São Paulo – USP, São Paulo, Brazil

**Keywords:** water maze, neurogenesis, epilepsy, pilocarpine, aging

## Abstract

Increased adult neurogenesis is observed after training in hippocampal-dependent tasks and also after acutely induced status epilepticus (SE) although the specific roles of these cells are still a matter of debate. In this study, we investigated hippocampal cell proliferation and differentiation and the spatial learning performance in young or aged chronically epileptic rats. Status was induced by pilocarpine in 3 or 20-month old rats. Either 2 or 20 months later, rats were treated with bromodeoxyuridine (BrdU) and subsequently underwent to 8-day schedule of water maze (WM) tests. As expected, learning curves were faster in young than in aged animals (*P* < 0.001). Chronically epileptic animals exhibited impaired learning curves compared to age-matched controls. Interestingly, the duration of epilepsy (2 or 20 months) did not correlate with the memory impairment of aged-epileptic animals. The number of BrdU-positive cells was greater in young-epileptic subjects than in age-matched controls. In contrast, cell proliferation was not increased in aged-epileptic animals, irrespective of the time of SE induction. Finally, dentate cell proliferation was not related to performance in the WM. Based on the present results we conclude that even though aging and epilepsy lead to impairments in spatial learning, their effects are not additive.

## Introduction

Temporal lobe epilepsy (TLE) is the most frequent type of epilepsy in adult humans (1). Most of TLE cases are characterized by the spread of complex partial seizures arising from limbic regions such as the hippocampus (1). Deficits in cognitive abilities, such as the disruption of declarative memory, are secondary to hippocampal dysfunction and seem to be progressive during spontaneous recurrent seizure (SRS) evolution (2, 3). It has been suggested that cognitive disruption may be due more to focal epileptic discharges than to lesions caused by status epilepticus (SE) (4). Greater cognitive losses have been reported in patients with long lasting chronic epilepsy (5, 6), with progressive cognitive decline in those with uncontrolled seizures (7). Thus, although these results could suggest that each spontaneous seizure could be implicated in cognitive decline, it remains to be clarified whether this can be dissociated from aging or not.

Pilocarpine-induced SE in rats is followed by several events that chronologically simulate those observed in the development of human TLE (8). Of special interest, Parent et al. (9) reported that SE acutely triggers neurogenesis of dentate granule cells. This phenomenon has since been studied in various animal models of TLE (10–15). Changes in cell proliferation rate were proposed to vary according to the progenitor cell vulnerability to each model of SE induction (16) and age of epilepsy onset (17). Although the physiological relevance of neurogenesis has not been fully demonstrated, granule cell proliferation in the dentate gyrus affects the formation of temporal associations during memory acquisition and retention (18). It is also known that the chronic epileptic condition (19–22), as well as aging, cause a decline in learning and memory capacities (23–26). On the other hand, memory dysfunction in humans correlates to the low capacity of dentate granule cells to proliferate and differentiate in elderly patients (27).

The attempts to establish a causal linkage between hippocampal neurogenesis and spatial learning performance has yielded conflicting results. While a positive correlation between hippocampal neurogenesis and the acquisition rate in Morris’ water maze (WM) task was shown for young adult mice (28), no such effect was observed for young (29) or aged (30) rats. The lack of consistency may be related to specificity of animal species, age, and breeding conditions, the fate of new generated neurons, bromodeoxyuridine (BrdU) injection time, and the respective analytic window. In order to contribute to above-mentioned observations, this study was designed to investigate the effects of aging and duration of epilepsy on the basal proliferation and survival rate of newborn dentate granule cells before animals are subjected to Morris’ WM.

## Materials and Methods

### Animals

Male Wistar rats (*n* = 42) were obtained from CEDEME-UNIFESP and housed in groups (five to six young rats or three aged rats) with free access to food and water. Rats were kept at controlled temperature, humidity, and light (∼22°C, 60% relative humidity, and a 12/12 light/dark cycle). All experimental protocols were approved by the Institutional Animal Care and Use Ethics Committee of the Universidade Federal de São Paulo (protocol number 1341/06) and were performed in accordance with the guidelines for animal research of the Society for Neuroscience.

### Experimental design

**Figure 1:**
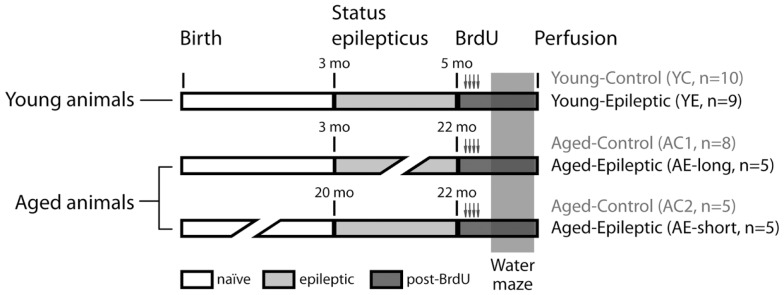
**Experimental groups**. Rats were treated with pilocarpine to induce status epilepticus, control rats received saline instead of pilocarpine. Bromodeoxyuridine (BrdU) was administered for 2 days, starting 9 days before perfusion. Spatial learning was evaluated by the latency to find a hidden platform in a water maze (WM). Production and survival of newborn cells in the subgranular zone of the granule cell layer were measured through counting of BrdU and doublecortin (DCX) labeled cells, 24 h after the last day of the WM task.

Rats were treated with pilocarpine as described below to induce SRS. Pilocarpine was administered at the age of either 3 months (*n* = 14) or 19–20 months (*n* = 5). Rats that received pilocarpine at 3 months of age were tested either 2 months later [young-epileptic (YE) group, *n* = 9, upper line in Figure 1, with 5 months of age] or between 19 and 20 months later [aged-epileptic group with long lasting epilepsy (AE-long), *n* = 5, with 22–23 months of age]. Rats that received pilocarpine at 20 months of age were tested 2 months later [aged-epileptic group with short lasting epilepsy (AE-short), *n* = 5, bottom line in Figure 1]. The observations (*n* values) for each group represent the total number of animals that survived after the pilocarpine treatment and that were used in the analysis. Thus, the two groups of aged subjects were tested at similar ages, i.e., 22–23 months old, but differed in the duration of their epileptic life. The two groups with short-time epilepsy (YE and AE-short) had the condition for 2 months. Three groups of age-matched control rats received intraperitoneal (i.p.) injections with saline instead of pilocarpine at corresponding ages (3 months; *n* = 18, ∼20 months; *n* = 5, see Figure 1). As there were no statistically significant differences between the two aged-control groups, their data were combined. The number of animals per group refers to those who reached the end of the experimental design. That is, each epileptic animal reported here had at least 5 min of uninterrupted seizures before being considered in SE. The seizure manifestation was clearly staged according to the Racine criteria described below; the behavioral manifestation of SE was attenuated 90 min after its onset. During behavioral test days, animals were video-recorded for 3 h prior and during tests. Those animals that had SRS within 2 h prior or during the WM task were excluded from analysis and not reported here to avoid confounding effects on WM performance during post-ictal state.

### Induction of status epilepticus

The protocol used for SE induction was published previously in detail (31, 32). Briefly, SE was induced by i.p. injection of pilocarpine hydrochloride (320 mg/kg, i.p. Merck, USA). To reduce activation of peripheral receptors by pilocarpine, scopolamine methyl bromide (1 mg/kg, i.p. Sigma, USA) was given 30 min prior to pilocarpine. Approximately 20 min after pilocarpine injection, young and aged animals started to develop motor seizures. Seizures were classified according to Racine’s kindled stages III (bilateral forelimb clonus), IV (stage III and rearing), and V (stage IV and loss of postural tone, i.e., rearing and falling) each one lasting 30–50 s (33), which progressed to SE. Young adult rats developed SE ∼40 min after pilocarpine treatment, whereas aged rats developed SE within 30 min of pilocarpine injection. To reduce SE severity and mortality, thionembutal (25 mg/kg, i.p. Cristalia, Brazil) was administered 90 min after the onset of SE (8). SE severity, based on behavioral analysis and mortality rates (∼20% after SE onset), did not differ between young and aged animals. SE rats received intensive care and were hand-fed for at least 3–4 days after SE to increase their survival rate. All animals used in this study developed SRS, which was measured by periodic video monitoring. As previously demonstrated by us and others in rats and non-human primates, the main feature of pilocarpine model is that SRSs observed during the long-term period resemble those of human complex partial seizures and recurs two to three times per week per animal (34–36).

### Spatial memory test

Spatial learning and memory was tested using the WM as described previously (37). The entire procedure was performed by one of the authors (CFC) to minimize inter-animal stress levels. Briefly, a black round fiberglass pool, 200 cm in diameter and 50 cm high, was filled to a depth of 25 cm with water (temperature: 26 ± 1°C). A transparent acrylic platform (9 cm diameter), mounted on a transparent support, was placed in the center of the northeast quadrant, 1–2 cm below water surface. The pool-room measured 3.05 m and had several salient visual cues on the walls.

In each swimming trial, a rat was placed in the water near one side of the pool facing the wall, at one of the following locations: south, southeast, east, or northeast, at random. The rat was allowed to swim until it found the hidden platform. If the rat did not find the platform within 120 s, it was manually guided to the platform and was left there for 30 s. The rat was then removed from the platform, dried thoroughly with a towel, and placed either into a waiting box until the next trial or, in its home cage after the last trial of the day.

All rats were subjected to a training phase that consisted of four swimming trials a day, ∼30 min apart, for seven consecutive days. During this training phase, the time needed to reach the platform decreased progressively. Twenty-four hours after the end of the training phase, the platform was removed, and the rats were randomly placed in the pool and allowed to swim for 60 s. The time spent in the northeast quadrant, where the platform was located earlier, was recorded.

The swim path was recorded at 10 Hz with a video camera positioned 290 cm above the center of the pool, and analyzed with an image analysis system (VP112, HVS Image Ltd., Hampton, UK) that computed the latency to reach the hidden platform, swim path, swimming velocity, and the time spent within each pool quadrant.

### Bromodeoxyuridine treatment

Bromodeoxyuridine was administered before the WM in all experimental groups (Figure 1). Rats received four i.p. injections of 50 mg BrdU per kg body weight. The injections were administered at 12 h intervals, starting 2 days before the beginning of the WM training sessions. The last BrdU injection was administered 16 h before the first training session.

### BrdU and doublecortin immunohistochemistry

Twenty-four hours after the final swimming session, animals were deeply anesthetized with thionembutal (50 mg/kg, i.p. Cristalia, São Paulo, Brazil) and perfused through the heart with 4% paraformaldehyde. Their brains were removed, postfixed in 4% paraformaldehyde for 2 h at 4°C, and cryoprotected in 30% sucrose in phosphate buffer (PB). Thirty micrometers thick coronal sections throughout the entire antero-posterior axis of the hippocampus were cut using a cryostat. Sections from each animal were sliced and collected sequentially in 24-well plates, cryopreserved in an anti-freezing solution before storage at −20°C.

The immunohistochemistry procedure for BrdU was defined *a priori* and it was performed on every 12th section of the entire hippocampus (distance between section ∼360 μm). With this sampling, we were able to run experiments on 1 out of 12 representative sections along the rostro-caudal extension of the dentate gyrus. Sections were incubated for 10 min with 3% H_2_O_2_ in PB, washed in PB, followed by 10 min in 1 N HCl. After treatment with blocking buffer (BB) solution, which consisted of 5% bovine fetal serum and 0.1% Triton X-100 in 0.1 M PBS, sections were incubated overnight with a monoclonal antibody to BrdU raised in rat (1:200, Axyll/Accurate Chemical, Westbury, NY, USA) diluted in BB. The sections were then washed and incubated with biotinylated anti-rat secondary antibody (1:500, Vector Laboratories, Burlingame, CA, USA) during 2 h in PB solution. The sections were stained with the Vectastain ABC Kit (Vector Laboratories, Burlingame, CA, USA) and the diaminobenzidine method. Stained sections were mounted on gelatin-coated slides, air-dried, dehydrated, cleared, and coverslipped with Entellan^®^ (Merck, Darmstadt, Germany).

Co-localization of BrdU and doublecortin (DCX) was examined in the sections adjacent to those used for BrdU labeling. The procedure included incubation with a mixture of the primary anti-BrdU antibody (described above) and anti-DCX (raised in rabbit, polyclonal, 1:500, Cell Signaling Technology, Danvers, MA, USA) overnight. The fluorescent secondary antibody for detection of BrdU was Alexa 488 conjugated anti-rat (1:1000; Molecular Probes, Eugene, OR, USA). Doublecortin was detected with a secondary anti-rabbit Alexa 546 conjugated (1:500; Molecular Probes, Eugene, OR, USA), both incubated in PBS during 60 min in the dark. Rinsed tissue sections were mounted onto gelatin-subbed slides and coverslipped in the dark using Vector Vectashield (Vector Laboratories, Burlingame, CA, USA). To evaluate the severity of hippocampal cell damage at different ages, coronal brain sections were processed for histopathological evaluation. Six sections per animal were selected (adjacent to those already used) for NeuN immunostaining. In brief, after treatment with BB, as described above, the sections were incubated overnight in polyclonal antibody to NeuN raised in mouse (1:1000, Millipore, Temecula, CA, USA) diluted in BB solution. Sections were washed and incubated with a biotinylated anti-mouse secondary antibody (1:500, Vector Laboratories) during 2 h in PB solution. Staining was produced with the Vectastain ABC Kit (Vector Laboratories, Burlingame, CA, USA) and the diaminobenzidine method. Stained sections were mounted on gelatin-coated slides, air-dried, dehydrated, cleared, and coverslipped with Entellan^®^ (Merck, Darmstadt, Germany).

### Cell counting

An experienced scorer (CFC), unaware to the experimental condition of slices, performed BrdU- and BrdU/DCX-positive cells manually. Since the number of proliferating cells labeled with BrdU and BrdU/DCX in the dentate gyrus was very low (specially in aged groups) and the spatial distribution of BrdU was inhomogeneous, we count the total (absolute) number of labeled cells in each of the 12 sections per animal (38). For this we took care to assure that each analyzed section was similar among all groups. BrdU-positive cells were counted bilaterally, visualized with a light microscope (Nikon Eclipse E600FN) using 20× objective and captured with a Nikon DXM 1200 digital camera. Cell counting is expressed as averaged number of labeled cells per group using total number of labeled cells in each animal.

Double staining for BrdU and DCX was analyzed in six coronal sections, adjacent to one set of sections chosen for the BrdU labeling procedure, of the dentate gyrus of each animal. Sections were observed with a confocal laser microscope (LSM 510, Zeiss, Oberkochen, Darmstadt, Germany) with argon (488 nm) and helium-neon (543 nm) lasers and a 40× oil immersion objective. As BrdU and DCX are localized in different compartments of the cell, co-localization analysis included visual inspection of size and shape of the cell through a stack of parallel planes. The BrdU/DCX co-localization was calculated by dividing the number of double-labeled cells by the number of BrdU-positive cells.

The number of hilus NeuN-positive cells was counted from each animal in six coronal sections parallel to the BrdU-labeled sections, from the both hemispheres. The hilar area under analysis was set at 0.0288 mm^2^ for each animal. The results are expressed as the number of hilar neurons/mm^2^.

### Statistical analysis

Results are presented as the means ± SEM. Behavioral performance on the WM was evaluated by changes in latency and path length to find the hidden platform. These scores were subjected to repeated-measures analyses of variance (ANOVA) with Age and Condition as the between-subjects factors, and Sessions and Trials as the within-subjects factors; a different ANOVA was used for each score. Comparisons for the time spent in the target quadrant on the eighth day, probe trial and the number of BrdU-, BrdU/DCX-, and NeuN-labeled cells in the hilus of the dentate gyrus was carried out using ANOVA with Age and Condition as the between-subjects factors. When required, the ANOVA was followed by a *post hoc* Student Newman–Keuls test. Relationships between spatial memory (as expressed by reduction of latency between the first and the last training days) and cell proliferation (BrdU/DCX-positive cells) were evaluated using Spearman’s rank-order correlation coefficient, *r_s_*. Significance was set at *P* < 0.05 (Bonferroni-adjusted significance level for multiple comparisons).

## Results

### Spatial memory performance in aged and/or epileptic animals

**Figure 2:**
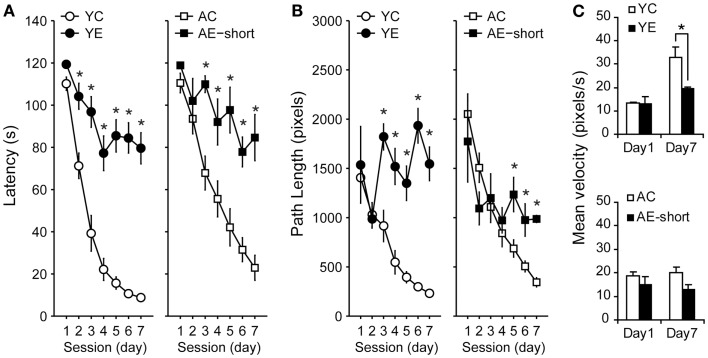
**Effect of pilocarpine-induced epilepsy on WM performance. (A)** Performance of rats 2 months after pilocarpine-induced SE, i.e., during the chronic phase. Values represent the average latency of four trials to reach a hidden platform (error bars indicate SEM). During the 7-days of training, all animals learned to locate the hidden platform (Repeated-Measures ANOVA). Control groups (white symbols) showed improved performance compared to the epileptic (black symbols) groups, despite their age. While young-control animals showed a steeper reduction in latency to find the hidden platform in the first 4 days, aged-matched controls improved monotonically during all training days. **(B)** Average path length as in **(A)**. The abscissa in **(A,B)** show training days; the ordinate shows the average time taken to reach the platform and path length, respectively, on four trials on each day. **(C)** Averaged speed velocity in the first (Day 1) and last (Day 7) days of training. YC, young control; YE, young epileptic; AC, aged control; AE-short, aged with status epilepticus at 18 months of age. *P < 0.05 compared to aged-matched controls (Student Newman–Keuls *post hoc* test).

Young and aged rats that were tested in the WM 2 months after pilocarpine-induced SE (YE and AE-short groups) or saline administration (YC and AC groups) reduced their latencies [Figure 2A, Repeated-Measures ANOVA, effect of sessions: *F*_(6,198)_ = 65.3, *P* < 0.001] and path length [Figure 2B, Repeated-Measures ANOVA, effect of sessions: *F*_(6,198)_ = 19.5, *P* < 0.001] to reach the hidden platform. Nevertheless, control and epileptic rats differed in their learning curves [ANOVA, interaction between condition and sessions: *F*_(6,198)_ = 11.5 and *F*_(6,198)_ = 6.5, *P* < 0.001, for latency and path length, respectively]. Training strongly reduced the latency to find the platform (Figure 2A) and increased swimming speed (Figure 2C) in non-epileptic young-control animals (YC group). Improvement of performance was less steep in young-epileptic subjects as compared to YC [ANOVA, effect of condition: *F*_(1,33)_ = 30.7, *P* < 0.001, for latency].

Young and aged-control rats had a less steep slope learning curve, i.e., latency reduction was more accentuated than its respective epileptic groups (Figure 2A). Aged animals showed slower learning curves than young condition-matched groups [interaction between age and condition: *F*_(1,33)_ = 7.5 and *F*_(1,33)_ = 27.2, *P* < 0.01, for latency and path length, respectively]. There was no significant interaction between sessions (i.e., days of training), experimental condition, and age [*F*_(6,198)_ = 1.0 and *F*_(6,198)_ = 1.1, *P* > 0.05, NS, for latency and path length, respectively]. By the end of the training sessions, young-epileptic rats took nine times longer than age-matched controls to reach the hidden platform, while aged-epileptic rats took three times longer than their age-matched controls (Figure 2A). Similar results were observed for path length. Young-control animals increased their average swimming speed while the young-epileptic group and both aged groups did not significantly increase their average swimming speed (Figure 2C). Changes in swimming speed may have contributed to reducing the latency curve of young-control animals (Figure 2A).

### Effects of untreated epilepsy on the spatial memory task

**Figure 3:**
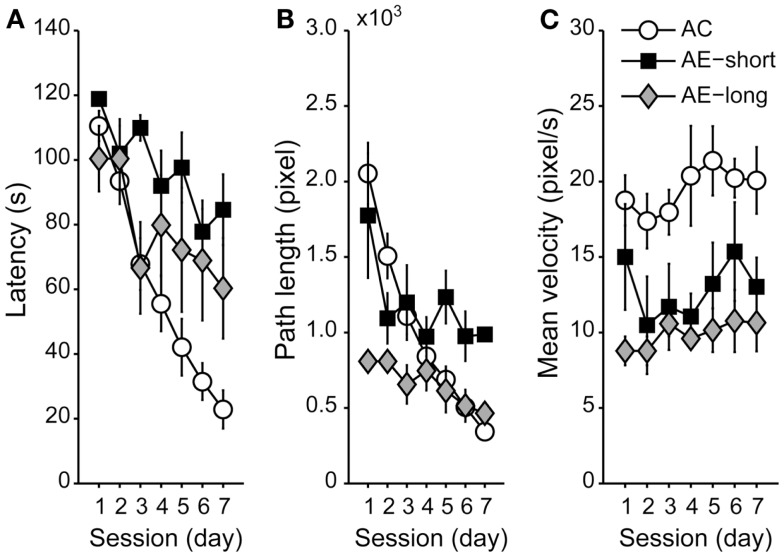
**Performance of aged animals in the WM shown as a mean of (A) latency, (B) path length, and (C) velocity to find the hidden platform, as in Figure 2**. Data are represented as the means ± SEM. All aged animals learned to find the hidden platform along the sessions (Repeated-Measures ANOVA), but epileptic animals performed worse than aged control (Student Newman–Keuls *post hoc* test). AC (white circle): aged controls; AE-short (black square): aged with status epilepticus at 20 months of age; and AE-long (gray diamond): aged with status epilepticus at 3 months of age.

To compare spatial memory between aged animals with a long versus a short course of untreated epilepsy, we compared aged animals injected with pilocarpine at the age of either 3 or 20 months. Aged animals reduced their latency and path length to the hidden platform along sessions [Figures 3A,B; *F*_(6,120)_ = 21.1 and *F*_(6,120)_ = 14.8, *P* < 0.05, for latency and path length, respectively]. *Post hoc* analyses revealed that aged-epileptic animals performed worse than aged controls, irrespective of the duration of the epileptic condition [effect of condition: *F*_(2,20)_ = 5.0 and *F*_(2,20)_ = 3.8, *P* < 0.05; Repeated-Measures ANOVA, for latency and path length, respectively] indicating that there is no effect of AE-short versus AE-long on the WM performance. Interestingly, aged animals did not improve their swimming speed during the training session unlike the young-control animals [effect of session: *F*_(2,20)_ = 0.9, *P* > 0.05, NS; Figure 3C]. Aged-epileptic animals swam slower in comparison to aged-control animals [effect of condition: *F*_(2,37)_ = 6.9, *P* < 0.01].

### Hippocampal cell proliferation in chronic epileptic rats

**Figure 4:**
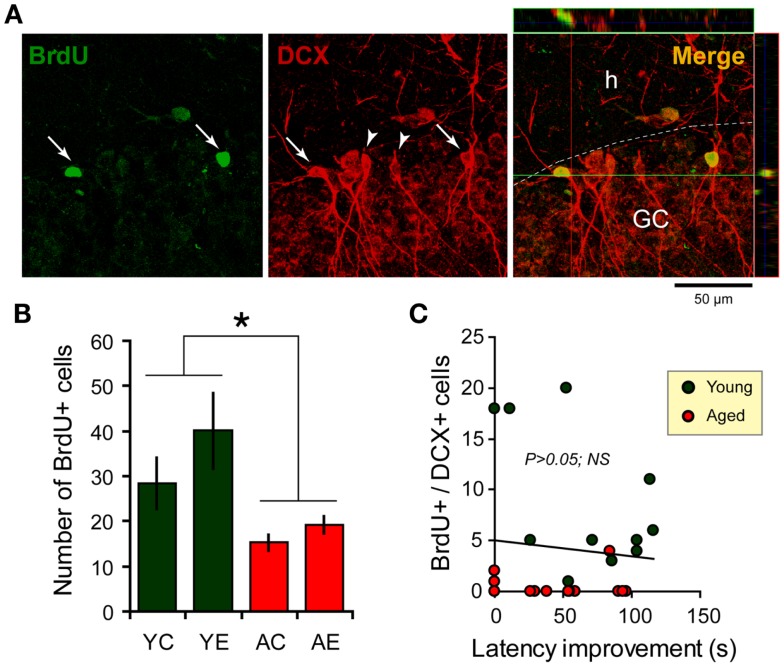
**Newborn cells in the dentate gyrus (DG) over the period of the spatial memory task as examined through bromodeoxyuridine (BrdU, green) and doublecortin (DCX, red) immunostaining**. **(A)** Newborn cells (arrows) in the DG co-express DCX (arrowheads), a marker of immature neurons. Flaps on the top and at the left of the main photograph show images merged from orthogonal planes that run through the red and green lines of the main photograph. **(B)** Quantification of BrdU-positive cells in the hippocampus shows an increase in the number of new cells added over a similar period of time for young-epileptic (YE) group compared to young controls (YC) and aged animals (AC, aged control; AE, aged epileptic). *P < 0.05 comparing young to aged groups (ANOVA, followed by SNK *post hoc* test). Values are expressed as the mean ± SEM. **(C)** No correlation was found between the number of BrdU+/DCX+ cells and the performance 7 days after training in the water maze (latency improvement). Data from young and aged animals are represented in different colors for clarity purposes (correlation curve not shown,*P* > 0.05).

The two-way ANOVA analysis of the number of BrdU-labeled cells indicated an effect of age: *F*_(1,31)_ = 11.2, *P* < 0.05, and an effect of condition: *F*_(1,31)_ = 5.8, *P* < 0.05; but there is no interaction between both factors. The dentate cell proliferation rate was higher in the younger than in aged rats. Young-epileptic animals tended to have higher number of BrdU-positive cells when compared to young controls. This difference was indeed effective when they were compared to aged animals with a later epilepsy onset (AE-short) or aged controls, as shown in Figure 4B. There were no differences in the number of BrdU-positive cells among aged animals in all groups, either long-term or short-term.

With respect to double-labeled cells (Figure 4A), the fraction of BrdU-labeled cells that expressed DCX was higher in older than in younger rats. In fact, almost all BrdU-labeled cells in older rats were also double stained for DCX. In younger rats, 64% of BrdU cells were double labeled in the control group and 71% in the epileptic group. The rate of newborn cells in younger rats was higher than in aged animals, as previously demonstrated (31).

There was no clear relation between behavioral performance in the WM and hippocampal cell proliferation in the dentate granule layer. The correlation between the number of BrdU and DCX double-labeled cells and the latency improvement was not significant (*R* = −0.0936; *P* > 0.05; *n* = 26; Figure 4C). Statistical correlation was not found either when considering young and aged animals separately (data not shown, although Figure 4C depicts both groups in different colors).

### Neuron loss in aged and epileptic animals

**Figure 5:**
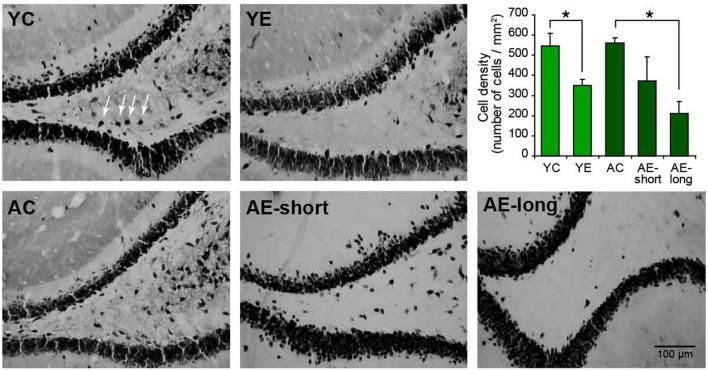
**Representative photomicrographs of the hilar region of the DG of the hippocampus stained for NeuN**. The graphic shows the quantification of NeuN-positive cells in the hilus. The young-epileptic group (YE) exhibited significant neuron loss compared to the young-control group. Among aged animals, the long epileptic life group (AE-long) also exhibited significant neuron loss as comparing to aged-control animals, but the short (AE-short) do not have a significant effect. *P < 0.05 comparing control to epileptic animals (ANOVA, followed by SNK *post hoc* test). Values are expressed as the mean ± SEM.

To verify the damage produced by epilepsy, aging, or both, NeuN-positive cells were quantified in the hilus of the dentate gyrus of the hippocampus, a region highly susceptible to injury (39). The two-way ANOVA revealed significant neuron loss in the hilar region of epileptic animals compared to controls [effect of condition: *F*_(1,18)_ = 7.3, *P* < 0.05]. The status induced in aged animals, causes highly variable cell loss within group (AE-short: 372.5 ± 113.4 versus YE: 342.6 ± 18.6), thus the number of remaining hilar cells in aged-epileptic animals was not statistically significant different from aged-control ones (560.9 ± 26.0). The AE-long group showed significantly decreased hilar NeuN-positive cells (210.7 ± 59.1) when compared to the AC group (*P* < 0.05; Figure 5).

## Discussion

The present study shows that both aging and epilepsy impair WM task acquisition. These factors are not additive but interrelated because the performance of epileptic animals was similar despite their age. Interestingly, new dentate cells increased significantly only in young animals and consequently, no correlation between dentate neurogenesis rate and WM performance in either controls or epileptic animals was observed. Rats with long or short lasting histories of epilepsy showed no gross differences in spatial task performance, although the present results may suggest that short-term memory was more affected in rats with long lasting epilepsy (Figure 3, Day 7). However, among aged animals, neuron loss was observed in the hippocampus only after a long epileptic life, but not after short-term epileptic life.

### Epilepsy and cognition

Our finding that aging leads to learning and memory deficits in a hippocampal-dependent task is in agreement with previous studies (40–43). One mechanism to explain such deficits is impaired experience-dependent place field expansion plasticity (44), which can lead to non-appropriate activity sequences of temporal order activity in aged animals. A series of studies has shown that repeated passage of young rats through a fixed path causes asymmetric expansion in the size of the hippocampal place field (44–46) in a sequence reactivation pattern that is proposed to occur via inherent asymmetry in the hippocampal network established by the CA3–CA1 Schaffer collateral synapse (47). In agreement with this hypothesis, a recent study demonstrated that the temporal order of activity patterns is poorly preserved in aged animals (48).

In accordance with the current findings, previous studies have demonstrated that young-epileptic pilocarpine-treated animals also display impaired performance in classical hippocampus-dependent tasks when compared to their controls (49–51). The additional novel finding reported here is that young and aged animals with a 2-month history of epilepsy displayed similar deficits, indicating that the aging-related deterioration of learning and memory capacity was not further compromised by the epileptic condition. This is consistent with the recent demonstration that spatial memory is selectively affected soon after the initial SE insult (52). A reduction in power and frequency of the theta rhythm before and after the behavioral task has been proposed to explain this change. A similar impairment of rhythmogenesis may also disturb memory encoding and retrieval (53) and is more evident in aged animals (54). Impairment of theta rhythm may account for the observed cognitive deficits observed in the epileptic rats in our study. In humans, failures in memory formation during encoding are related to reduced entorhinal-hippocampal coherence and decreased theta oscillations (55, 56). Although the mechanisms that cause impaired learning and memory in the epileptic condition are not entirely clear, the above-mentioned studies may explain why the epileptic condition is able to exacerbate the poor cognitive performance of aged animals.

Consistent with earlier reports in humans and animals, the current findings support the notion that chronic epilepsy affects cognition (1). Long-term studies that addressed cognitive impairment in epilepsy have produced variable results, with some suggesting impaired cognitive ability (5, 6), others suggesting stable neuropsychological functioning (57), and yet others suggesting progressive cognitive decline with ongoing seizures, with recovery from deficits when seizures cease (58). The comparison between epilepsy models that promote intense or mild neuronal injury (i.e., kainate versus kindling in amygdala) suggests that neuronal injury is the central factor related to cognitive impairment in TLE. Human data suggest substantial cognitive deficits at the onset of epilepsy, which stabilize during the first 5–10 years of epileptic life [for review, see (3)]. These long-term studies support the idea that progressive, epilepsy-associated decline in cognitive function is strongly related to uncontrolled seizures. The current findings, comparing aged animals with long or short duration epilepsy and hilar cell loss, did not show any conclusive evidence that the injury-based explanation or the alternative seizure frequency has a central role in the impaired cognition of epileptic rats, even though age differences within each aged group might have contributed to increased variability in the spatial performance and cell proliferation. On the other hand, when comparing young-epileptic versus aged-epileptic (AE-short) groups, our results strongly suggest that a cognitive deficit develops rapidly after SE and that little further decline occurs later (AE-long). Nevertheless, we are aware that the inclusion of additional aged, epileptic chronic animals would help to strength the conclusions of the present study.

### Epilepsy, cell proliferation, and differentiation in young and aged chronic epileptic animals

Formation of new granule cells in the dentate gyrus occurs in rodents, primates, and humans (59, 60). Some of these new cells may differentiate into neurons and become part of the hippocampal circuitry forming new synapses with already existing neurons (61– 64). It has been suggested that these neurons improve hippocampus-dependent learning and memory formation (27, 65), although other studies failed to find a contribution of these cells to these cognitive functions (29, 66) we did not analyze the proliferation rate in animals that were not subject to WM. Based on the difficulties to obtain aged-epileptic animals, we prioritize to analyze the animal in all possible aspects. Furthermore, Van der Broght and colleagues had already described that WM has no effect on hippocampal neurogenesis on male adults Wistar and Sprague-Dawley rats (29). The lack of correlation between dentate granule cell proliferation and performance in the WM task observed in our experiments, either in epileptic or in controls, also suggests that the functional significance of these newborn cells is low. It is important to emphasize that the lack of correlation was observed when using data from young and aged animals separately. This analysis is important to avoid confounding effects due to “floor effect” (i.e., when no correlation can be established because of very low or absent proliferating cells) Therefore, although the effects of WM on cell proliferation cannot be completely ruled out, its effects on our results are unlikely. Actually, it was previously shown that hippocampal neurogenesis is not affected by training male adults rats in the WM (29). In addition, if WM induce neurogenesis in the present experimental protocol, we would expect a general increase in cell proliferation and differentiation in all groups, fostering differences between groups with different proliferative capabilities. Finally, cell proliferation in aged animals was extremely low and if WM has an effect, if any, would be to increase BrdU+ staining and not to decrease it, leading to an enhanced difference between young and aged groups.

Most dentate gyrus cells in rats are produced near the end of the first postnatal week. Generation continues at a lower rate throughout adulthood (67), and dramatically falls with aging (19, 68). Our finding that aged rats were less able to produce newborn cells than young animals is consistent with these studies.

Doublecortin is a cytoskeletal protein that is transiently expressed only in newborn neurons, widely used as a marker of neuron progenitors. While several studies have shown that neurogenesis in the dentate gyrus increases after acute seizures and SE in young rats (9, 12, 16, 69, 70), seizure-induced neurogenesis is not as robust in aged animals (71, 72). In all these studies, the rate of proliferation was measured soon after seizure onset. In the current study, cell proliferation was measured 2 months after SE in young and aged animals following the hippocampal-dependent WM task. In agreement with a previous report (17, 31), we found that DCX-positive cells were almost absent from the dentate granule cells of aged controls and aged rats with late epilepsy onset. Similarly, hippocampal tissue from patients with late-onset, drug-resistant, TLE showed reduced neurogenesis (27). Taken together the decreased proliferation rate and the absence of BrdU cells not stained for DCX in aged animals suggest that not only aged rats have fewer newborn neurons but also show no proliferation of glial cells. These data are in accordance with Arisi et al. (73), who found no significant alteration in astrocyte density in the hippocampus of aged rats analyzed 1 month after pilocarpine-induced SE. We can conclude that aged rats, epileptic or not, showed almost no newborn cells in the hippocampus after the WM task.

Finally, since the hippocampus is highly susceptible to injury (39), cognitive performance in aged, epileptic animals may be affected by the extend of cell loss alone. We have previously shown a significant decrease in CA1 pyramidal cells in the hippocampus of both, young and aged, epileptic animals (32). Here we extended this result and showed that it is the epileptic condition, and not the age, that affect cell number and survival in the hilus of the hippocampus. Based on this we suggest that the neuronal loss and reduced cell proliferation in aged-epileptic animals might contribute to memory impairment associated with this pathological condition.

### Study limitation

As above-mentioned, in the current study, we did not analyze the proliferation rate in animals that were not subject to WM. This was mainly limited by the difficulties to obtain aged-epileptic animals and to have an acceptable number of animals free of spontaneous seizures in close temporal proximity to the WM trial that had completed the entire experimental protocol. Therefore, we prioritized to analyze these animals in all possible aspects. The above-mentioned restriction limits our capacity to evaluate the roles that potential newly generated neurons might have had in hippocampal-dependent learning and memory formation among aged animals. The fact that the neurogenesis rate is so low among aged groups, lead us to suppose that the basal proliferation and differentiation of dentate granule cells have low relationship to the poor task performance. The same restricted number of animals in such experimental conditions is also likely responsible for the low but significant power of the statistical analysis.

## Conclusion

In summary, our results show important changes in neurogenesis in the rat hippocampus caused by aging and chronic epilepsy. The untreated epileptic condition and natural aging are related to hippocampus-dependent memory dysfunction, but when both conditions are present, no additional impairment is observed. Thus, it could be suggested that both aging and epilepsy share mechanisms. On the other hand, it could also be suggested that at least two mechanisms are converging on the same response and are not additive once each mechanism alone causes a maximal response. Thus, it is possible that epilepsy or aging, when present alone, impair learning by separate mechanisms and that their combination leads to the same level of maximal impairment. The spatial task induced faster learning curves in aged-control animals than in aged-epileptic ones, but this was not related to dentate proliferation/differentiation of newborn cells. Thus, under the current experimental conditions, neurogenesis in epileptic animals did not seem related to better learning and memory, regardless of age.

## Conflict of Interest Statement

The authors declare that the research was conducted in the absence of any commercial or financial relationships that could be construed as a potential conflict of interest.
